# Cerebral hemodynamics of the aging brain: risk of Alzheimer disease and benefit of aerobic exercise

**DOI:** 10.3389/fphys.2014.00006

**Published:** 2014-01-21

**Authors:** Takashi Tarumi, Rong Zhang

**Affiliations:** ^1^Institute for Exercise and Environmental Medicine, Texas Health Presbyterian Hospital DallasDallas, TX, USA; ^2^Department of Internal Medicine, University of Texas Southwestern Medical CenterDallas, TX, USA; ^3^Department of Neurology and Neurotherapeutics, Alzheimer's Disease Center, University of Texas Southwestern Medical CenterDallas, TX, USA

**Keywords:** cerebral hemodynamics, aging, Alzheimer's disease, vascular dementia, regular aerobic exercise

## Abstract

Alzheimer disease (AD) and cerebrovascular disease often coexist with advanced age. Mounting evidence indicates that the presence of vascular disease and its risk factors increase the risk of AD, suggesting a potential overlap of the underlying pathophysiological mechanisms. In particular, atherosclerosis, endothelial dysfunction, and stiffening of central elastic arteries have been shown to associate with AD. Currently, there are no effective treatments for the cure and prevention of AD. Vascular risk factors are modifiable via either pharmacological or lifestyle intervention. In this regard, habitual aerobic exercise is increasingly recognized for its benefits on brain structure and cognitive function. Considering the well-established benefits of regular aerobic exercise on vascular health, exercise-related improvements in brain structure and cognitive function may be mediated by vascular adaptations. In this review, we will present the current evidence for the physiological mechanisms by which vascular health alters the structural and functional integrity of the aging brain and how improvements in vascular health, via regular aerobic exercise, potentially benefits cognitive function.

## Introduction

Alzheimer's disease (AD) is a devastating neurological disorder characterized by progressive deterioration of brain structure and function (Querfurth and LaFerla, 2010). Advanced age is the strongest risk factor for AD such that the risk doubles every 5 years after age of 65. Due to the rapid aging of the population, the prevalence of AD is facing an exponential growth. However, there remains a dearth of effective treatments, cures, and most importantly preventions (Thies and Bleiler, 2013).

Mounting evidence indicates that vascular disease and risk factors not only elevate the risk of vascular dementia (VaD) but also AD (de la Torre, 2004). Traditional views on the etiology of AD and VaD have been divergent. AD develops as a result of brain amyloid depositions, leading to a pathological cascade of neurodegeneration and cognitive impairment (Selkoe, 1991; Hardy and Selkoe, 2002). On contrary, VaD is attributed to cerebral hypoperfusion and ischemia that are associated with impairment of synaptic activity and protein synthesis, glutamate excitotoxicity, and neuronal apoptosis (Hossmann, 1994; Gorelick et al., 2011). Despite these traditional perspectives, large population-based prospective studies have demonstrated that vascular risk factors in midlife, such as systolic hypertension and hyperlipidemia, led to a significant elevation of AD risk in later life (Kivipelto et al., 2001).

Vascular risk and dysfunction are modifiable through lifestyle modifications (Ornish et al., 1990). In particular, physical activity has been shown to improve cognitive outcomes in patients with mild cognition impairment (Lautenschlager et al., 2008). In contrast to the pharmacological interventions, habitual physical activity such as “regular aerobic exercise” is low cost, has virtually no adverse effects, and can be important for primary prevention of AD (Selkoe, 2012). Nonetheless, the physiological benefits of regular aerobic exercise on AD prevention are not completely understood and are only supported by a limited amount of data. Accordingly, the primary objective of this review is to overview the current evidence of the association between vascular aging and AD pathology. Secondarily, we will extend our discussion to the potential mechanism by which regular aerobic exercise may attenuate AD pathology via improvements in vascular health.

## Cerebrovascular anatomy and blood flow regulation

The brain relies critically on a constant supply of blood due to its high rate of oxidative metabolism and lack of energy substrate. ~15% of cardiac output is directed to the brain which only weighs ~2% of body mass yet accounts for ~20% of total blood glucose and oxygen utilizations (Attwell et al., 2010). To sustain the high volume blood supply, cerebrovascular resistance is low. However, this makes the brain sensitive to changes in cerebral blood flow (CBF) during hypo- or hypertension (Faraci and Heistad, 1990). Importantly, the majority of cerebrovascular resistance is controlled outside of the parenchyma by extracerebral arteries (i.e., large cerebral arteries and pial arterioles) while intracerebral arterioles and capillaries account for the remaining resistance (Faraci and Heistad, 1990). Therefore, the coordinated adjustment of extra- and intracerebral arteries in response to changes in perfusion pressure is crucial in maintaining adequate perfusion and normal brain function.

Cerebral autoregulation (CA) maintains CBF relatively constant in the face of changes in arterial pressure (Lassen, 1959; Paulson et al., 1990). In particular, extracerebral adjustment of arteriolar resistance ensures adequate perfusion to the parenchyma (Rowbotham and Little, 1965; Faraci and Heistad, 1990). Watershed areas of deep and periventricular white matter are particularly vulnerable to hypoperfusion and hypoxia, especially with age-related vascular disease and dysfunction (Rowbotham and Little, 1965; Faraci and Heistad, 1990; Matsushita et al., 1994). Dysregulation of CBF and perfusion pressure may cause ischemic brain injuries such as white matter hyperintensities (WMH) and silent infarcts which are often associated AD pathology (Snowdon et al., 1997). Below we will discuss steady-state and dynamic regulations of CBF and its implications on the aging brain and AD pathology.

### Regulation of steady-state CBF

CBF decreases with advancing age and is lower in patients with AD compared with age-matched healthy individuals (Leenders et al., 1990; Matsuda, 2001). Age- and/or AD-related reductions in CBF are, at least in part, explained by the concurrent atrophy or lower metabolic rate of brain tissue (Matsuda, 2001). However, the recent evidence demonstrating the association between vascular disease and a higher incidence of AD also raises a potential hypothesis that dysregulation of CBF may cause an insufficient supply of energy and nutrients and accelerate the AD pathology (de la Torre, 2004). In healthy individuals, CBF and metabolism are tightly coupled via the neurovascular unit which regulates microvascular resistance upon neuronal activities (Attwell et al., 2010). In contrast, in patients with amnestic mild cognitive impairment, a prodromal stage of AD, global CBF and metabolic rate of oxygen were reduced and cerebrovascular resistance was elevated when compared with the normal controls (Liu et al., 2013). Furthermore, a linear relation that was observed in healthy subjects between global CBF and metabolic rate of oxygen was absent in patients with amnestic mild cognitive impairment. These findings suggest the presence of neurovascular decoupling of CBF and metabolism in the individual who is at greater risk of AD. Pathologically, cerebral hypoperfusion or hypoxic ischemia have been shown to increase amyloid plaque depositions, the pathological hallmark of AD, which precedes neurodegeneration and cognitive impairment (Zhang et al., 2007; Okamoto et al., 2012).

Physiological mechanisms by which vascular disease and risk factors promote the AD pathology are likely to be multi-factorial and related to a number of changes in vascular structure and function (Breteler, 2000). Although the relative degree to which each component of vascular abnormalities plays a role remains unclear, endothelial dysfunction, a hallmark of vascular aging, has been observed in patients with AD (Dede et al., 2007). Vascular endothelium, the most internal layer of the arterial wall, is susceptible to blood-derived chemical and mechanical stimuli. In response to these stimuli, the endothelium releases vasoactive substances to maintain vascular homeostasis. In particular, constitutive nitric oxide (NO) plays a crucial role in maintaining vasomotor tone and its dynamic regulation facilitates functional hyperemia in response to changes in metabolic demand (Fujii et al., 1991; Dietrich et al., 1996). Importantly, NO also inhibits atherogenesis by reducing oxidative modification of low-density lipoprotein (LDL) cholesterol and preventing the proliferation of vascular smooth muscle cells (Davignon and Ganz, 2004). Oxidation of LDL has been proposed as a major mechanism of the atherosclerotic process (Davignon and Ganz, 2004). In patients with AD, age-related impairment of endothelial function is exacerbated such that endothelium-dependent NO-mediated vasodilatory function, as assessed by brachial flow-mediated dilation, is lower when compared with healthy subjects (Dede et al., 2007). Consistently, exposure of cerebrovascular endothelium to amyloid-β (Aβ) peptide acutely impairs endothelium-dependent vasodilation by augmenting oxidative stress (Thomas et al., 1996). Moreover, autopsy studies have demonstrated a greater atherosclerotic burden in patients with AD than healthy controls (Roher et al., 2003). Collectively, these findings suggest that cerebral endothelial dysfunction, Aβ induced vasoconstriction, and atherosclerotic encroachment of cerebral arteries may elevate cerebrovascular resistance leading to brain hypoperfusion which in turn may accelerate the AD pathology.

AD and atherosclerosis also share the common genetic risk factors, such as the ε4 allele of apolipoprotein E (APOE4) (Casserly and Topol, 2004). Apolipoprotein E is a polymorphic protein arising from three alleles (i.e., ε 2, ε 3, and ε 4), which differs by only a single amino acid substitute. Yet these changes have the profound influences on the brain and peripheral lipid metabolism (Mahley and Rall, 2000). Although the underlying cellular and molecular mechanism remains to be established, APOE4 is associated with greater Aβ depositions in the brain (Bu, 2009; Kim et al., 2009) and higher plasma concentrations of LDL. Increased LDL elevates the risk of atherogenesis, especially when associated with endothelial dysfunction (Wilson et al., 1994, 1996).

### Regulation of dynamic CBF: beat-to-beat low frequency oscillations

The brain is susceptible to dynamic changes in arterial pressure which spontaneously oscillates at the low frequencies below heart rate at rest (Zhang et al., 2000). CA during dynamic beat-to-beat changes in arterial pressure more or less is a frequency dependent phenomenon which may dampen oscillations of CBF in response to changes in arterial pressure at the low frequencies (Lassen, 1959; Paulson et al., 1990; Zhang et al., 1998). CA is an inherent property of cerebral arteries and arterioles that are controlled by myogenic, neurogenic, and metabolic mechanisms. In effect, CA ensures a constant supply of oxygen and nutrients during hypotension while attenuating hyperperfusion during hypertension.

Contrary to the general pattern of age-related impairment of vascular function, CA appears to remain intact in older adults (van Beek et al., 2008). Furthermore, recent studies suggest that CA is also preserved in patients with AD (Claassen et al., 2009; Zazulia et al., 2010), although the presence of cerebral amyloid angiopathy, which often accompanies advanced AD pathology, may alter CA. Cerebral amyloid angiopathy is characterized by the accumulation of Aβ_1−40_ proteins in the cerebral vessel wall (Christie et al., 2001). Aβ_1−40_ is highly toxic to cerebral vasculature such that that exogenous application of Aβ_1−40_ impairs endothelial-dependent vasodilation and exaggerates vasoconstriction (Thomas et al., 1996). Using a transgenic mouse model of AD, profound impairment of CA has been observed (Niwa et al., 2002). Of note, the inconsistent findings between the preclinical and clinical studies of CA in AD clearly warrants future studies to determine whether CA is indeed impaired in AD and whether changes in CA are related to the AD onset or progression.

AD may also impair cerebral tissue oxygenation during dynamic changes in CBF. Cerebral tissue oxygenation, measured non-invasively be near-infrared spectroscopy, predominantly reflects the oxygen saturation of venous blood (Madsen and Secher, 1999; Claassen et al., 2006; Rowley et al., 2007; Murkin and Arango, 2009). In patients with AD, transfer function gain of cerebral tissue oxygenation in response to changes in CBF has been reported lower while the phase was reduced when compared with controls subjects (van Beek et al., 2010). These findings suggest that more arterial oxygen is transmitted to the venous circulation and a possibility of microvascular dysfunction in which the brain tissue is less able to extract oxygen from the arterial blood. If this is the case, it can be speculated that neurovascular coupling of CBF and metabolism may be disrupted during hemodynamic challenges in patients with AD.

### Regulation of dynamic CBF: cardiac frequency

The human heart is an intermittent pump which generates a stroke volume every cardiac cycle. During healthy youth, left ventricular afterload, as well as systolic blood pressure generated to overcome the afterload, is low (Avolio et al., 1983). In addition, the compliance of central arteries effectively dampens the hemodynamic pulsations via the Windkessel effect and creates a continuous blood flow in the microcirculation of peripheral vascular beds (Nichols et al., 2005). With advancing age, central elastic arteries stiffen and total peripheral resistance increases. As a result, left ventricular afterload increases and so does systolic blood pressure (Avolio et al., 1983).

Advancing age is also associated with a premature timing of arterial wave reflection. In the circulatory system with arterial branching and tapering, a forward-traveling pressure wave generated from the left ventricle encounters the discontinuity of vascular impedance/resistance and reflects back a portion of the incident waves (Nichols et al., 2005). With the age-related increase in aortic pulse wave velocity, a reflected pressure wave collides prematurely with the incident pressure waves, thus leading to the augmentation of systolic and attenuation of diastolic pressures (Nichols et al., 2005). As discussed above, dynamic CA is effective in dampening changes in arterial pressure only at low frequency (<0.1 Hz) (Zhang et al., 1998). Therefore, age-related increase in hemodynamic pulsatility at cardiac frequency may be transmitted passively (i.e., without the counteraction of CA) into the brain and lead to brain structural damage.

Cerebrovascular impedance is elevated in the elderly while the brain is exposed to a greater magnitude of hemodynamic pulsatility (Zhu et al., 2010). Vascular impedance represents an opposition to pulsatile blood flow and is determined by the intrinsic property (i.e., elasticity) and diameter (i.e., resistance) of blood vessels (O'Rourke and Taylor, 1967). Age-related increases in cerebrovascular impedance with the concurrent elevations in CBF pulsatility observed in the basal cerebral arteries may reflect a compensatory mechanism of cerebral vasculature which attenuates the transmission of CBF pulsatility into the delicate microcirculation. In support of this, vascular adaptations to hemodynamic pulsatility have been shown from the peripheral vasculature (Laurent et al., 2009). For example, central arterial stiffness and higher systolic blood pressure were positively associated with thicker wall of resistance vessels relative to the lumen diameter (Muiesan et al., 2013). Moreover, greater central pulse pressure was positively associated with wall-to-lumen ratio of retinal arterioles which is an independent risk factor for stroke (Wong et al., 2001; Ott et al., 2013). Such adaptations of the microvasculature increase vascular impedance which would attenuate the transmission of hemodynamic pulsatility, reduce vascular damage, and facilitate oxygen extraction. However, these adaptations may occur at the expense of increases in vascular resistance and thus reduction in steady-state brain perfusion. Indeed, central arterial stiffness is negatively correlated with CBF in the deep white matter where a high prevalence of WMH are observed (Tarumi et al., 2011). Moreover, central arterial stiffness and pressure pulsatility are associated with higher prevalence of subcortical infarct, atrophy of brain parenchyma, and greater levels of brain amyloid plaques (Mitchell et al., 2011; Hughes et al., 2013; Nation et al., 2013). These findings collectively indicate the potential importance of age-related changes in central hemodynamics to cerebrovascular remodeling and structural brain changes. In addition, prevention of central arterial aging may alleviate pulsatile-induced cerebral microvascular disease and age-related cognitive decline.

## Aerobic exercise and the brain structure and function

There is an increasing recognition that habitual aerobic exercise enhances cognitive function and attenuates age-related deterioration of brain structure. Earlier studies in animals showed the greater benefits of voluntary aerobic exercise in improving cognitive function when compared with other stimuli such as expanded learning opportunities (van Praag et al., 1999). Furthermore, exercise-related improvements in cognitive function were associated with the neurogenesis of hippocampal dentate gyrus in the adult mouse (van Praag et al., 1999). Human studies also demonstrated that regular walking increased the hippocampal size and improved the memory performance in the previously sedentary elderly individuals (Erickson et al., 2011).

Regular aerobic exercise preserves the structural integrity of white matter (Gons et al., 2013; Tseng et al., 2013). As assessed by diffusion tensor MR imaging, Master's athletes, who have participated in a lifelong high intensity, high volume aerobic exercise training, attenuated age-related reductions in axonal fiber integrity, as shown by higher fractional anisotropy and lower mean diffusivity in the network of front-to-back connections (Tseng et al., 2013). Moreover, there was a strong positive correlation between maximal oxygen uptake and fractional anisotropy in the left superior longitudinal fasciculus (Tseng et al., 2013). These findings suggest that regular aerobic exercise preserves the microstructural integrity of white matter that is responsible for visuospatial function, motor control, and coordination (Tseng et al., 2013).

Exercise-related improvements in brain function and structure may be conferred by the concurrent adaptations in vascular function and structure. Aerobic exercise increases the peripheral levels of growth factors (e.g., BDNF, IFG-1, and VEGF) which cross the blood-brain barrier (BBB) and stimulate neurogenesis and angiogenesis (Trejo et al., 2001; Lee et al., 2002; Fabel et al., 2003; Lopez-Lopez et al., 2004). Consistent with this, exercise-related enlargement of hippocampus was accompanied by increases in cerebral blood volume and capillary densities (Pereira et al., 2007). Enhanced cerebral perfusion may not only facilitate the delivery of energy substrates, but also lower the risk of vascular-related brain damages, including WMH and silent infarct (Tseng et al., 2013). Furthermore, regular aerobic exercise is associated with lower levels of Aβ deposition in individuals with APOE4 positive (Head et al., 2012), which may also reduce the risk of cerebral amyloid angiopathy and microbleeds (Poels et al., 2010).

Regular aerobic exercise ameliorates endothelial dysfunction and central arterial stiffness (Seals et al., 2008). As discussed above, central arteries serve as an important interface between the cerebral and peripheral circulations (Nichols et al., 2005). A recent study which compared middle-aged endurance-trained and sedentary adults demonstrated that higher aerobic fitness in endurance-trained adults was correlated positively with better cognitive performance and negatively with central arterial stiffness, independent of other lifestyle factors (e.g., sleep and diet) (Tarumi et al., 2013). Furthermore, lower central arterial stiffness, as assessed by carotid artery distensibility, showed positive associations with cognitive performance and resting CBF in the occipito-parietal area (Tarumi et al., 2013). In addition, exercise-related enhancement of endothelial function may facilitate neurovascular coupling and protect cerebral arteries from atherogenesis. In humans, endothelium-dependent vasodilatation measured from the peripheral (e.g., brachial and radial) arteries is considered a systemic index of vascular endothelial function (Celermajer et al., 1994; DeSouza et al., 2000). If this is also the case for cerebral circulation, exercise-related improvement in endothelial function is likely to benefit CBF regulation, structural integrity of BBB, and cognitive outcome. Currently, there is a relative lack of data as to the impact of exercise-related improvement in endothelial function on brain structure and function. Future studies are needed to confirm whether a reversal of endothelial dysfunction translates into better brain structural and cognitive outcomes.

## Summary

AD and cerebrovascular disease often coexist in the aging brain. Traditional view that AD and VaD are two divergent clinical entities with few or no overlap between their pathologies is challenged by an accumulating body of evidence indicating a close association between vascular disease and the risk of AD development. More recently, physiological studies also have demonstrated that vascular dysregulation of CBF elevate the risk of both cerebrovascular disease and AD. Specifically, presence of central arterial stiffness and endothelial dysfunction elevates the risk of pressure pulsatility or flow-related damage to brain structure and function. Further study of these modifiable risk factors through pharmacological or non-pharmacological approaches is likely to be important for developing an effective prevention or treatment for AD. In particular, further mechanistic study of the effect of regular aerobic exercise on arterial aging, brain perfusion and structure may provide new insights into how to prevent or slow the age-related cognitive decline, AD, and other age-related cerebrovascular diseases.

## Author contributions

Takashi Tarumi drafted the manuscript. Rong Zhang revised the manuscript critically for important intellectual content. Rong Zhang and Takashi Tarumi approved the final version of the manuscript.

### Conflict of interest statement

The authors declare that the research was conducted in the absence of any commercial or financial relationships that could be construed as a potential conflict of interest.
